# Application of Fractions of Crop Evapotranspiration Affects Carbon Partitioning of Grapevine Differentially in a Hot Climate

**DOI:** 10.3389/fpls.2021.633600

**Published:** 2021-02-22

**Authors:** Nazareth Torres, Runze Yu, Johann Martínez-Lüscher, Evmorfia Kostaki, Sahap Kaan Kurtural

**Affiliations:** Department of Viticulture and Enology, University of California, Davis, Davis, CA, United States

**Keywords:** biomass partitioning, deficit irrigation, photosynthesis, primary metabolism, sugar allocation, water scarcity

## Abstract

Majority of viticulture regions are located in mid-latitudes characterized by weather variability and stressful environments relying on irrigation for mitigating environmental stress during the growing season and to ensure a profitable yield. The aim of this study was to characterize the response of grapevine (*Vitis vinifera* L. cv. Cabernet Sauvignon) to different applied water amounts based on the replacement of fractions of crop evapotranspiration (ET_c_) during two growing seasons with contrasting precipitation patterns. The experiment consisted of three irrigation treatments based on the weekly replacement of 25, 50, and 100% of ET_c_. Grapevine stem water potential decreased during the growing season reaching its lowest value (-1.5 and -1.2 MPa, respectively) at harvest in the more stressed vines (25 and 50% ET_c_). Leaf gas exchange variables were measured during the two seasons and 100% ET_c_ had the highest rates of photosynthesis and stomatal conductance and better instantaneous water use efficiency, also resulting in higher leaf chlorophyll and carotenoid content. Mineral nutrient content for nitrogen and potassium increased linearly with the increase in applied water. At harvest, no differences were observed in the number of clusters per vine; however, the 25% ET_c_ had the lowest berry size and yield per vine with no difference in sugar content of berry. Conversely, sugar allocation to reserve organs was highly affected by applied water leading to different shoot to root biomass partitioning, where shoot:root ratio, leaf non-structural carbohydrates, and photosynthetic pigments increased with greater applied water. Likewise sucrose:N ratio and root non-structural carbohydrates decreased with the lower applied water. Altogether, carbon allocation between the source and sink organs likely controlled the response of grapevines to water deficits in a hot climate, and replacing 50% ET_c_ was sufficient to sustain the grapevine performance given the enhancement of sugar transport, which could slow down the detrimental effect of water deficits on yield.

## Introduction

Within perennial crops, the grapevine (*Vitis vinifera* L.) is the most economically important fruit crop with more than 7.4 million cultivated hectares worldwide in 2016 (OIV, 2016). Many of the viticulture areas of the world rely on irrigation for consistent production (Rienth and Scholasch, 2019). In the last decades, there is an increasing need for irrigation within the traditionally non-irrigated regions due to the current permanent rise in global air temperature and higher evapotranspiration with no appreciable gain in precipitation (Costa et al., 2016). Furthermore, the majority of viticultural regions are forecasted to experience a reduction in cloud coverage and rainfall and an increase in solar radiation reaching the earth’s surface (Trenberth and Fasullo, 2009), leading to higher temperatures and, consequently, higher evaporation from soils.

Micro-irrigation strategies aim to replace frequently just the amount of water to meet the actual crop evapotranspiration (ET_c_) demand in the immediacy of the root zone without using the storage capacity of the soil (Allen et al., 1998). Irrigation of vineyards usually introduces a predetermined water deficit. Therefore, deficit irrigation has emerged as a potential strategy to allow grapevine to withstand water shortage during the growing season without yield loss and maintaining the berry composition (Chaves et al., 2010; Costa et al., 2016). Severe restrictions of water availability may accelerate sugar accumulation in grape berry in hot climates (Bonada et al., 2015; Zarrouk et al., 2016) and result in adverse effects on yield (Nelson et al., 2016), fruit composition (Brillante et al., 2018), or wine composition (Yu et al., 2020). However, a sustained moderate water deficit may improve canopy microclimate, increase water use efficiency, control vigor, and reduce berry size improving berry quality by means of enhancement of sugars and flavonoids in red wine grape (Santesteban et al., 2011; Zarrouk et al., 2012; Cook et al., 2015; Martínez-Lüscher et al., 2020). Nevertheless, the final berry composition and consequent wine quality are highly dependent upon the proper control of carbohydrate partitioning, which balance the growth and metabolism of the source:sink organs (Yu et al., 2020).

Non-structural carbohydrates (NSC) are responsible for providing energy and carbon for grapevine growth, being stored as reserves in grapevine perennial organs. The role of stored NSC in the early season is crucial until bloom when leaf photosynthesis becomes the primary source of carbon (Zapata et al., 2004). The capacity of grapevines for replenishing these carbohydrate reserves increases at mid-ripening (Candolfi-Vasconcelos et al., 1994). In addition, sugars directly or indirectly control a wide range of physiological processes, including photosynthesis, sugar transport itself, nitrogen uptake, defense reactions, secondary metabolism, and hormonal balance (Smeekens et al., 2010; Dai et al., 2014). Not only sugar transport and partitioning play key roles in the regulation of plant development, but also they influence how grapevines respond to biotic and abiotic stress factors (Meteier et al., 2019). NSC storage may be altered by both abiotic factors and internal competition for carbon in grapevine, and in turn, this may modify grapevine growth, yield, and berry chemistry (Holzapfel and Smith, 2012). Therefore, to ensure sufficient vegetative growth, yields, and acclimation to environmental stresses, grapevines must efficiently allocate available annual resources to both vegetative and reproductive tissues. Increased soil temperature due to the changing climate, especially before *veraison*, strongly affects seasonal balance between shoot and root growth, bloom, plant water use, photosynthesis, and the availability of carbohydrate reserves (Field et al., 2020). Water availability is a determining factor for cell growth and photosynthesis (Medici et al., 2014) and for the redistribution of carbohydrates between the source and sink organs (Lemoine et al., 2013). Indeed, shifting in root to shoot growth in response to external resource availability allows plants to minimize some critical resource limitations (Grechi et al., 2007). However, our understanding of the factors determining carbon allocation among the different organs remains limited. Additionally, the incidence of water deficits is particularly acute during fruit development, when there is a great competition for photoassimilates among newly established sinks such as flowers, seeds, lateral shoots, and fruit and permanent structures such as trunks, stems, and roots (Lemoine et al., 2013).

The objective of this study was to characterize the primary metabolism response of grapevines to different applied water amounts based on the replacement of fractions of ET_c_ on grapevine physiology, as well as to assess their effect on carbon partitioning among the source and sink organs during two growing seasons.

## Materials and Methods

### Plant Material and Experimental Design

The experiment was conducted during two consecutive seasons (2018–2019 to 2019–2020) in Oakville, CA (38.428 N, 122.409 W), with row orientation NW–SE. The vineyard was planted in 2011 with Cabernet Sauvignon (clone FPS08) on 110R rootstock at a spacing of 2.4 × 2.0 m (row × vine). The grapevines were trained to a bilateral cordon on a vertical shoot positioned trellis with a cordon 96 cm high above the vineyard floor and pruned to one-bud spurs. All other cultural practices, including vineyard fertilization, were standard for the area and conducted before treatment application. The experiment was designed as a randomized block with a one-way arrangement of the following fractions of ET_c_ replacement treatments: (i) 25% ET_c_, (ii) 50% ET_c_, and (iii) 100% ET_c_, with six replicates each consisting of five experimental units, three of which were used for data collection and the two on distal ends were treated as border plants. Plants were irrigated weekly with two drip emitters per vine.

### Irrigation Treatments

The irrigation treatments were applied by varying water application rate based on calculated ET_c_. Vineyard ET_c_ was calculated using the following equation: ET_c_ = ET_o_ × *K*_c_, where ET_o_ is reference evapotranspiration and *K*_c_ is the crop coefficient. During the experiment, evaluation of vineyard *K*_c_ over the course of the growing season was calculated using the shade cast beneath grapevines grown with 100% ET_o_ application from April 1 and the VSP-specific equation developed by Williams (2014) and adjusted for row spacing.

The irrigation treatments were imposed by varying the emitter output per vine in the drip line, and the irrigation pump was scheduled to run based on the application rate of the 100% ET_c_ treatment plots. Thus, by reducing the emitter output from 8 to 2 L/h and 4 L/h per emitter, respectively, the 25 and 50% ET_c_ plots necessarily received water at a fraction of 100% ET_c_ plots. The irrigation treatments started in April of each year. Harvest commenced when the berry total soluble solids reached ca. 24°Brix on average in all treatments on 25 September 2019 (114 DAF) and 8 September 2020 (115 DAF), respectively.

### Weather Conditions

Weather data (Table 1) were obtained from the California Irrigation Management Information System, CIMIS, station (#77, Oakville, CA, United States) located 160 m from the experimental vineyard (CIMIS, 2020). Daily ET_o_ was obtained using a modified version of the Penman–Monteith equation (Snyder and Pruitt, 1992). The number of days with temperatures above 30°C was counted for the 2018–2019 and 2019–2020 growing seasons.

**TABLE 1:** Weather conditions during the growing seasons of 2018–2019 and 2019–2020.

	Month													
		October	November	December	January	February	March	April	May	June	July	August	September	
Year		**Mean daily temperature (°C)**												Mean
2018–2019		15.8	11.4	9	9.7	7.5	11	15.4	14.6	19.7	19.6	20.8	19.2	14.5
2019–2020		15.4	11	9.5	8.8	11.4	10.7	14.6	17.4	19.7	19.2	21.1	20	14.9
		**Minimum daily temperature (°C)**												Mean
2018–2019		7.2	3.7	3.5	4.7	2.7	4.9	8.8	8.4	11.2	11.1	12.3	9.7	7.4
2019–2020		4.9	3.3	5.7	3.5	3.7	4.4	7.1	8.8	10.4	10.1	12.3	11.1	7.1
		**Maximum daily temperature (°C)**												Mean
2018–2019		26.4	21.3	15.3	15.9	12.9	17.5	23.3	22.4	29.2	29.9	31.2	29.4	22.9
2019–2020		26.6	20.8	14.3	15.4	20.6	17.6	23	26.2	29.5	30.2	31.8	31.4	24.0
		**Days with temperature over 30°C (no.)**												Total
2018–2019		2	2	0	0	0	0	3	0	11	13	19	13	63
2019–2020		7	0	0	0	0	0	3	8	11	14	17	20	80
		**Precipitation (mm)**												Total
2018–2019		36.3	135.0	77.5	248.5	422.2	145.6	12.5	88.9	0.0	0.2	0.0	1.5	1,168.2
2019–2020		0.2	24.4	66.0	58.5	1.0	29.8	25.9	26.1	0.2	0.2	1.6	0.3	234.2
		**Reference ET (ET_o_, mm)**												Total
2018–2019		97.3	53.1	38.0	35.3	40.1	82.1	133.7	189.5	190.3	189.4	174.9	138.5	1,362.1
2019–2020		115.3	56.8	23.8	37.5	81.6	84.1	132.9	163.0	197.3	194.0	169.1	126.7	1,385.1
		**m^3^/ha**												Total
2018–2019	25% ET_c_	0.0	0.0	0.0	0.0	0.0	0.0	0.0	0.0	146.5	267.3	186.7	178.1	778.6
	50% ET_c_	0.0	0.0	0.0	0.0	0.0	0.0	0.0	0.0	292.9	534.4	373.1	356.2	1,556.6
	100% ET_c_	0.0	0.0	0.0	0.0	0.0	0.0	0.0	0.0	585.8	1,068.8	746.5	712.5	3,113.6
2019–2020	25% ET_c_	0.0	0.0	0.0	0.0	0.0	0.0	0.0	30.2	156.3	170.6	159.6	74.2	590.9
	50% ET_c_	0.0	0.0	0.0	0.0	0.0	0.0	0.0	60.6	312.7	341.3	319.2	147.9	1,181.7
	100% ET_c_	0.0	0.0	0.0	0.0	0.0	0.0	0.0	121.3	625.4	682.3	638.5	295.8	2,363.3

*Weather data were obtained from the CIMIS weather station #77 (Oakville, CA, United States) located at the research site.*

### Plant Water Status and Leaf Gas Exchange Measurements

Plant water status was monitored by measuring the midday stem water potential (SWP) throughout both growing seasons every 2 weeks. A fully expanded leaf from each treatment-replicate, sun-exposed, and without sign of disease or damage from the northeast side of the canopy was selected and measured. Two hours prior to taking the measurements, foil-lined zip bags were placed on sun-exposed leaves and sealed before excising the petiole in order to suppress transpiration. SWP was then directly determined with a pressure chamber (Model 610 Pressure Chamber Instrument., PMS Instrument Co., Corvallis, OR, United States).

Leaf gas exchange was measured with a CIRAS-3 portable infrared gas analyzer system (PP Systems, Amesbury, MA, United States) featuring a broad-leaf chamber with a 4.5-cm^2^ window. For each date and experimental unit, three measurements were made ca. solar noon (11:30 to 13:30 h) on a healthy leaf under light-saturating conditions (>1,500 μmol m^–1^ s^–1^) and values were averaged. The cuvette was oriented perpendicularly to sunlight, which was always in saturating conditions (average of internal PAR = 1,969 ± 135 μmol m^–2^ s^–1^). Measurements were taken at 40% relative humidity, a CO_2_ concentration of 390 μmol CO_2_ mol^–1^, and using a flow to the chamber of 300 ml min^–1^.

To summarize the temporal information for plant water status and leaf gas exchange, the area under the curve for all the parameters was calculated by using natural cubic splines for both years individually and collectively. The resultant values were divided by the number of the days between the first and the last day of measurements in each year, and then normalized by the first measurement in the 2018-2019 season and 2019-2020 season to make the data comparable to each individual measurement.

### Petiole Mineral Nutrient Content

In June of each growing season (ca. anthesis), 50 leaves per treatment-replicate were collected, leaf blades were removed, and petioles were dried at 70°C in a forced air oven. Then, mineral nutrient analysis was carried out using inductively coupled plasma-mass spectrometry by Dellavalle, Inc., Fresno, CA, United States, as reported elsewhere (Yu et al., 2020). Total nitrogen (N) was determined *via* automated combustion analysis (method B-2.20), while phosphorus (P), potassium (K), sodium (Na), calcium (Ca), magnesium (Mg), zinc (Zn), manganese (Mn), boron (B), iron (Fe), and cuprum (Cu) were analyzed *via* nitric/perchloric acid digestion (method B-4.20) as described by Gavlak et al. (1994). Previous studies indicated that petioles and leaf blades are equally effective to predict mineral deficiencies (Schreiner and Scagel, 2017).

### Total Chlorophyll (*a* + *b*) and Carotenoid Contents

At mid-ripening of the second growing season, two leaves of each treatment-replicate were collected and 25 mg of tissue was used for determining total chlorophylls (*a* + *b*) and total carotenoids according to Sesták et al. (1971). Extraction was conducted by immersing the samples of fresh tissue in 5 ml of 96% ethanol at 80°C for 10 min. Absorbance of the extracts at 470, 649, 665, and 750 nm were determined with a spectrophotometer (Cary 100, Santa Clara, CA, United States). Then, total chlorophylls and total carotenoids were calculated by using the extinction coefficients and equations described by Lichtenthaler (1987) and expressed as mg/g of dried weight (DW).

### Yield Components and Total Biomass of Woody Parts

At harvest, 60 berries per treatment-replicate were randomly collected and weighed to determine berry mass. Then, in the 2019–2020 season, these berries were crushed for must sugar determination. Grapevines were harvested and clusters were counted and weighed on a top-loading balance. Total leaf area was calculated by defoliating one grapevine per treatment-replicate after harvest (early November 2019 and October 2020, respectively) and using the regressive relationship between leaf dry mass and leaf area. A subsample of oven-dried leaves (30 mg) from each treatment-replicate was collected for sugar and starch analysis. Then, leaf area to fruit ratio was calculated by dividing the leaf area by the yield per vine and reported as m^2^/kg.

Six weeks following the harvest of 2020 (15–19 October 2020), one grapevine per treatment-replicate was removed from the vineyard by using a mechanical spade. The grapevine was then portioned into trunk and cordon, roots, and stems (shoots). Then, each portion was weighed on a top-loading balance to obtain the fresh biomass of the portions. A subsample of shoots and fine roots was collected for organ-dried biomass estimation and sugar and starch analysis. Harvest index (ratio of yield to biomass) was calculated after oven drying the samples.

### Carbohydrate Extraction and Total Soluble Sugars and Starch Determination

Subsamples of leaves, shoots, and roots were oven-dried at 70°C to a constant weight. Dried tissues were ground with a tissue lyser (MM400, Retsch, Germany). Thirty milligrams of the resultant powder was extracted in ethanol:water (75:25) solution. Briefly, 1.5 ml was added to each sample and extracted for 10 min at 90°C in a water bath. Then, they were centrifuged at 10,000 rpm for a minute, and the supernatant was collected for sugar determination. The procedure was then repeated for starch determination in which the resultant pellet was used.

Total soluble sugars (SS) and individual sugars were determined in the shoot, leaf, and root ethanolic extracts and in the diluted berry must samples (1:10). Samples were filtered with PTFE membrane filters (diameter: 13 mm; 0.45 μm; CELLTREAT Scientific Products, Pepperell, MA, United States) and transferred into high-performance liquid chromatography (HPLC) vials and subjected to reversed-phase HPLC analysis. The equipment consisted of an Agilent 1100 system coupled to a diode array detector (DAD) and an Infinity Refractive Index Detector (RID) (Agilent Technologies Inc., Santa Clara, CA, United States). The reversed-phase column was Luna Omega Sugar (150 × 4.6 mm, 3 μm particle size, Phenomenex Inc., Torrance, CA, United States) with a guard column of 5 mm. The temperature of the column compartment was maintained at 40°C and the RID flow cell was kept at 35°C. The mobile phase consisted of isocratic elution with acetonitrile:water (v/v, 75:25) at a flow rate of 1.0 ml/min with a run time of 22 min. Standard solutions of 10 mg/L of D-glucose, D-fructose, D-sucrose, and D-raffinose were injected to obtain the retention time for each compound, and detection was conducted by RID. Sugar standards were purchased from VWR International (Radnor, PA, United States). Sugar concentration of each sample was determined by comparison of the peak area and retention time with standard sample curves.

Starch content of the roots, shoots, and leaves was conducted using the Starch Assay Kit SA-20 (Sigma-Aldrich, St. Louis, MI, United States) in accordance with the manufacturer’s instructions. Briefly, pellets of different tissues were dissolved in 1 ml DMSO and incubated for 5 min in a water bath at 100°C. Starch digestion commenced with the addition of 10 μl α-amylase and then incubated in boiling water for another 5 min. Then, the ddH_2_O was added to a total volume of 5 ml. Next, 500 μl of the above sample and 500 μl of starch assay reagent were mixed and incubated for 15 min at 60°C. Negative controls with the starch assay reagent blank, sample blank, and glucose assay reagent blank and positive controls with starch from wheat and corn were performed. Reaction started with the incubation of 500 μl of each sample and 1 ml of glucose assay reagent at 37°C and was stopped with the addition of 1 ml of 6 M sulfuric acid after 30 min. The reaction was followed with a Cary 100 Series UV-Vis Spectrophotometer (Agilent Technologies Inc., Santa Clara, CA, United States) and starch content was expressed as percent of starch per tissue dried weight.

### Statistical Analyses

Statistical analyses were conducted with R studio version 3.6.1 (RStudio: Integrated Development for R., Boston, MA, United States) for Windows. Seasonal integrals of SWP and gas exchange variables for each growing season and for both seasons were calculated by using the same software. All data were subjected to Shapiro–Wilk’s normality test. Data were normally distributed and subsequently submitted to an analysis of variance (ANOVA) to assess the statistical differences between the different irrigation amounts. For seasonal integrals, a two-way ANOVA was applied to assess the effect of the growing season (year) and irrigation amounts on SWP and gas exchange parameters. For all data, means ± standard errors (SE) were calculated, and when the *F* value was significant (*p* ≤ 0.05), Duncan’s new multiple range *post hoc* test was executed using “agricolae” 1.2-8 R package (de Mendiburu, 2016). Percentage data were transformed, according to the suggestion of the most likelihood test, into arcsine root square before ANOVA. Pearson correlation analyses were performed with the same software by using the “corrplot” package (Wei and Simko, 2017).

## Results

### Grapevine Mineral Nutrient Content, Water Status, and Gas Exchange Parameters of Cabernet Sauvignon Vines Subjected to Different Replacements of ET_c_

Weather data for the 2019 and 2020 growing seasons are shown in Table 1. Compared with the 2019 growing season, 2020 had 17 days more with temperature over 30°C, a maximum daily temperature of 1.1°C higher, and almost 800 mm less of precipitation, leading to an ET_o_ of 23 mm higher. On the other hand, the lower available water for grapevine growth resulted in smaller canopy development decreasing the ET_c_, which explained the lower irrigation amount of 2020 compared with 2019 (Table 1).

Petiole mineral nutrients were not affected by irrigation amounts in the 2018–2019 growing season (Supplementary Table S1). Conversely, total N increased in 100% ET_c_, while the K content in 25% ET_c_ vines decreased in the 2019–2020 growing season. The micronutrients were not affected by the applied water amounts in either year of the study.

**FIGURE 1:**
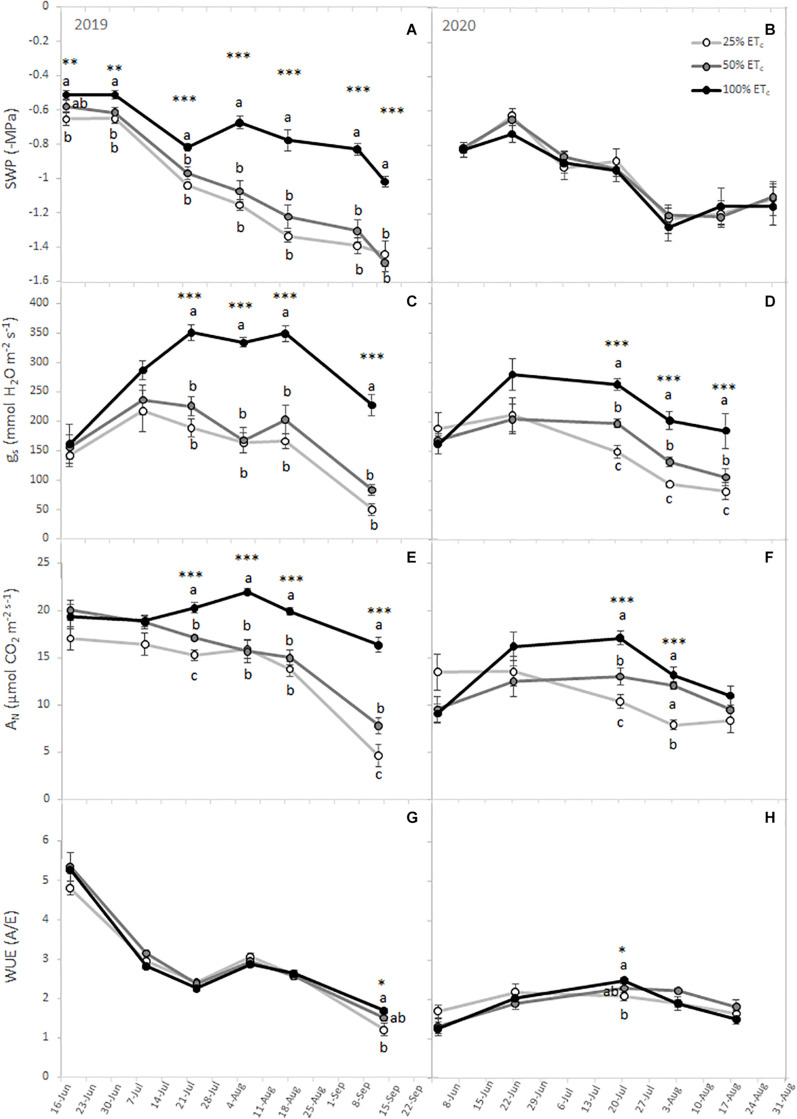
Stem water potential (SWP) **(A,B)**, leaf stomatal conductance (*g*_s_) **(C,D)**, leaf net carbon assimilation (*A*_N_) **(E,F)**, and intrinsic water use efficiency (WUE) **(G,H)** from Cabernet Sauvignon grapevines (clone FPS08), subjected to different replacements of crop evapotranspiration (25% ET_c_, 50% ET_c_, and 100% ET_c_) and collected through the 2019 and 2020 growing seasons in Oakville, CA, United States. Values represent means ± SEM (*n* = 6). At each time point, different letters indicate significant differences (*p* ≤ 0.05) between irrigation treatments according to one-way ANOVA followed by Duncan’s new multiple range test. *, **, and *** indicate significance at 5, 1, and 0.1 % probability levels, respectively.

The plant water status decreased throughout the season (Figures 1A,B). In 2019, the 100% ET_c_ treatment had the highest SWP, while 25% ET_c_ had the lowest SWP as expected. Conversely, there were no significant differences during the 2020 season between treatments. Likewise, we measured significant differences between the different irrigation amounts in *g*_s_ and *A*_N_ in both growing seasons (Figures 1C–F). We measured higher *g*_s_ and *A*_N_ in grapevines subjected to 100% ET_c_ treatment from the second half of July, coinciding with the *veraison*, to harvest, compared with 25% ET_c_. The *g*_s_ and *A*_N_ of 50% ET_c_ were transiently lower than those of 100% ET_c_, but consistently greater than those of 25% ET_c_. The WUE differed between irrigation amounts at harvest in 2019 and at mid-ripening in 2020 with 100% ET_c_ grapevines showing the highest WUE (Figures 1G,H). The enhancement of the photosynthetic performance in 100% ET_c_ grapevines was accompanied by increased total chlorophyll and carotenoid content in the leaves (Table 2).

**TABLE 2:** Yield components of Cabernet Sauvignon grapevines (clone FPS08) subjected to different replacements of crop evapotranspiration (25% ET_c_, 50% ET_c_, and 100% ET_c_), collected in Oakville, CA, United States in the 2018–2019 and 2019–2020 seasons.

	Clusters per vine (no.)	Yield (kg/vine)	Leaf area to fruit ratio (m^2^/kg)	Berry mass (g)
**2019**				
**Treatments**				
25% ET_c_	56 ± 1	6.78 ± 0.45^c^	0.472 ± 0.021^b^	1.08 ± 0.04^c^
50% ET_c_	58 ± 1	8.83 ± 0.44^b^	0.409 ± 0.029^b^	1.20 ± 0.04^b^
100% ET_c_	59 ± 1	10.35 ± 0.38^a^	0.726 ± 0.024^a^	1.37 ± 0.03^a^
ANOVA	ns	***	***	***
**2020**				
**Treatments**				
25% ET_c_	55 ± 1	4.80 ± 0.31^c^	0.499 ± 0.037^b^	0.85 ± 0.05^b^
50% ET_c_	53 ± 1	6.26 ± 0.32^b^	0.341 ± 0.013^c^	1.08 ± 0.02^b^
100% ET_c_	53 ± 1	9.14 ± 0.25^a^	0.838 ± 0.047^a^	1.15 ± 0.02^a^
ANOVA	ns	***	***	***

Values represent means (n = 6) separated by Duncan’s new multiple range test (p ≤ 0.05). Different letters within each column indicate significant differences as affected by the different irrigation amounts. ns and *** indicate non-significance or significance at 0.1% probability levels, respectively. DW, dried weight.

**FIGURE 2:**
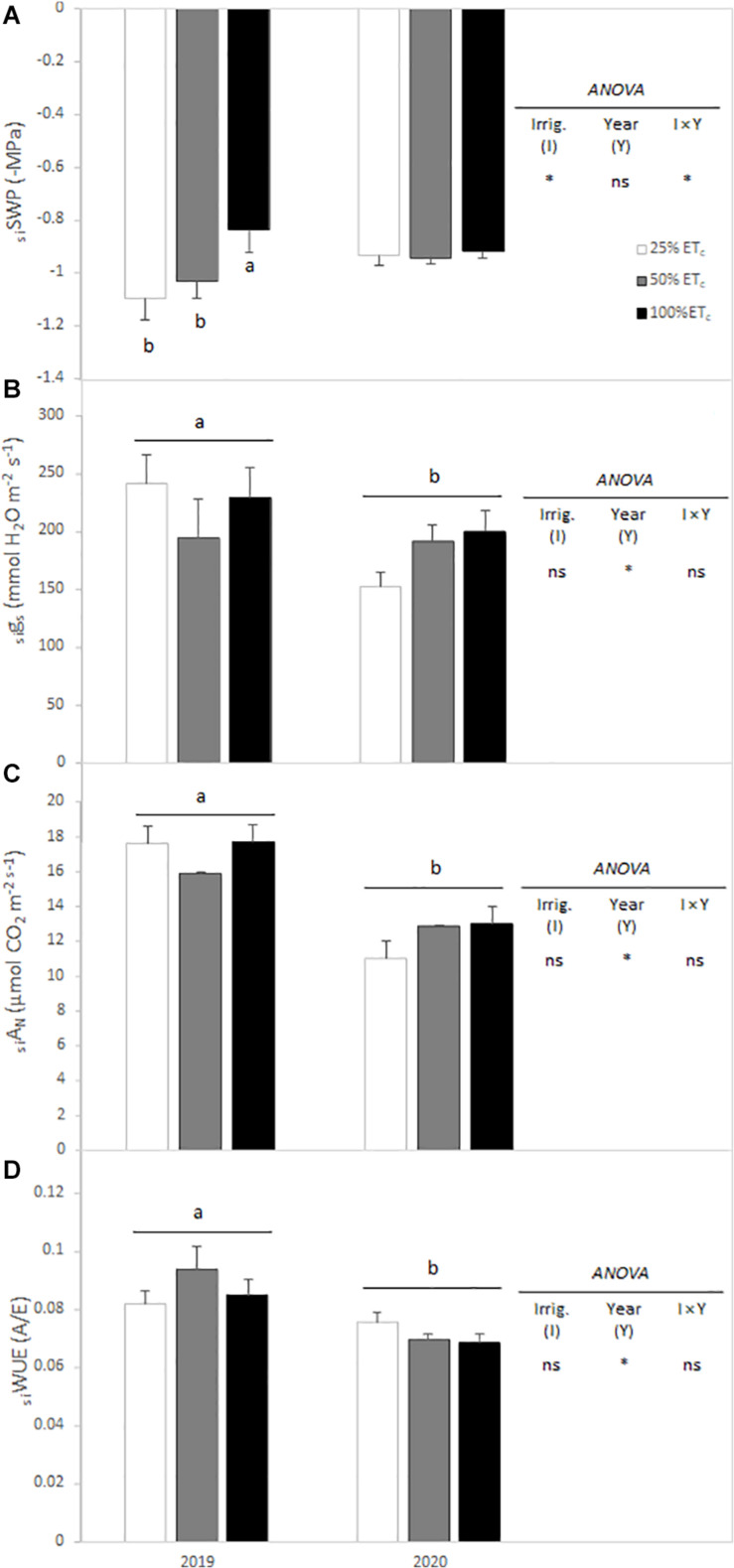
Seasonal integrals of stem water potential (_si_SWP) **(A)**, stomatal conductance (_si_g_s_) **(B)**, leaf net carbon assimilation (_si_A_N_) **(C)**, and water use efficiency (_si_WUE) **(D)** from Cabernet Sauvignon grapevines (clone FPS08), subjected to different replacements of crop evapotranspiration (25% ET_c_, 50% ET_c_, and 100% ET_c_) for the 2019 and 2020 growing seasons in Oakville, CA, United States. Values represent means ± SEM (*n* = 6). Different letters indicate significant differences (*p* ≤ 0.05) between irrigation treatments and year according to two-way ANOVA followed by Duncan’s new multiple range test. ns and * indicate non-significance or significance at 5% probability level, respectively.

Calculation of the seasonal integral of SWP and gas exchange variables allowed to establish the seasonal-long trend for grapevine physiological response. Thus, SWP seasonal integrals (_si_SWP) for both seasons were affected by the interaction between irrigation amount and year. During the 2019 season, there was a significant increase of SWP with 100 and 50% ET_c si_SWP compared with 25% ET_c si_SWP (Figure 2A). However, in the 2020 growing season, no difference in seasonal pattern was measured. On the other hand, seasonal integrals of *g*_s_, *A*_N_, and WUE were significantly different between years. The *A*_N_ and WUE were significantly lower in 2020 compared with 2019 (Figures 2B–D).

### Replacing Fractions of ET_c_ Modulated Yield Components and Vegetative Growth of Cabernet Sauvignon Grapevines

Grapevine growth was monitored for different organs as shown in Table 3 and Supplementary Table S2. Leaf, shoot, and root fresh weights increased with increased irrigation amounts (50 and 100% ET_c_ in 2019 and 100% ET_c_ in 2020, Supplementary Table S2). The biomass of the leaves, roots, and shoots increased in the grapevines subjected to 100% ET_c_ irrigation compared with 50 and 25% ET_c_ (Table 3). The applied irrigation treatments affected the harvest index (Table 3). The greatest harvest index was measured in 100% ET_c_, while the lowest was measured in 25% ET_c_, respectively.

**TABLE 3:** Total biomass (DW) of trunks, leaves, shoots, and roots; harvest index (yield to total biomass); and total chlorophylls (*a* + *b*) and total carotenoids of Cabernet Sauvignon grapevines (clone FPS08) subjected to different replacements of crop evapotranspiration (25% ET_c_, 50% ET_c_, and 100% ET_c_) during two growing seasons (2018–2019 and 2019–2020) and harvested in Oakville, CA, United States in November 2019 and October 2020, respectively.

	Leaves (kg/vine)	Shoots (kg/vine)	Trunk (kg/vine)	Roots (kg/vine)	Harvest index (kg/kg)	Shoot:root ratio	Total chlorophylls (mg/g DW)	Total carotenoids (mg/g DW)
**Treatments**								
25% ET_c_	0.39 ± 0.03^b^	0.42 ± 0.06^b^	2.52 ± 0.19	0.97 ± 0.04^b^	1.13 ± 0.10^c^	0.48 ± 0.08	2.36 ± 0.70^b^	1.15 ± 0.18^b^
50% ET_c_	0.47 ± 0.04^b^	0.57 ± 0.06^b^	2.89 ± 0.17	1.13 ± 0.10^ab^	1.26 ± 0.13^b^	0.50 ± 0.04	2.58 ± 0.47^b^	1.29 ± 0.16^b^
100% ET_c_	0.81 ± 0.07^a^	0.84 ± 0.20^a^	3.09 ± 0.22	1.35 ± 0.13^a^	1.51 ± 0.15^a^	0.65 ± 0.03	5.59 ± 0.27^a^	2.05 ± 0.10^a^
ANOVA	***	***	ns	*	***	ns	***	**

Values represent means (n = 6) separated by Duncan’s new multiple range test (p ≤ 0.05). Different letters within each column indicate significant differences as affected by the different irrigation amounts. ns, *, **, and *** indicate non-significance or significance at 5, 1, and 0.1% probability levels, respectively. DW, dried weight.

Cluster number was not affected by the replacement of different fractions of ET_c_ (Table 2). An increase in the yield per grapevine was observed in both seasons with a highly significant increase in yield per grapevine in 100% ET_c_ treatment. Likewise, the linear increase in yield was evident from 25% ET_c_ to 50% ET_c_ as well, in both years. We also measured linear increases in leaf area to fruit ratio and berry size as the amount of irrigation increased from 25% ET_c_ to 100% ET_c_.

### Carbohydrate Metabolism in Different Grapevine Organs Was Affected After Two Seasons of Different Irrigation Amounts

**FIGURE 3:**
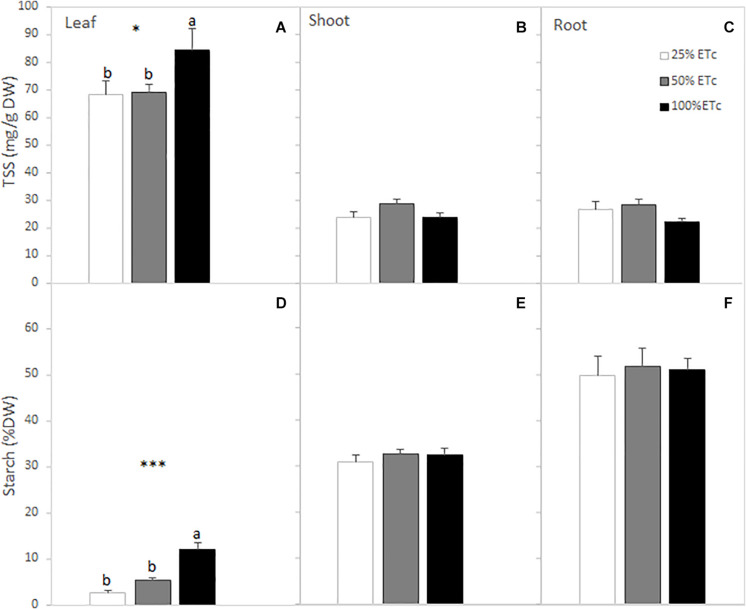
Leaf, shoot, and root total soluble sugar (SS) **(A–C)** and starch **(D–F)** contents of Cabernet Sauvignon grapevines (clone FPS08), subjected to different replacements of crop evapotranspiration (25% ET_c_, 50% ET_c_, and 100% ET_c_) during two growing seasons (2019 and 2020) and collected in October 2020 in Oakville, CA, United States. Values represent means ± SEM (*n* = 6). Different letters indicate significant differences (*p* ≤ 0.05) between irrigation treatments according to one-way ANOVA followed by Duncan’s new multiple range test. * and *** indicate significance at 5 and 0.1% probability levels, respectively.

There was a significant increase of SS and starch content in the leaves as affected by the applied water amount (Figures 3A,D). This increase in leaf SS was attributed to the increases in glucose, fructose, and raffinose content of the leaves (Table 4). The total sugar and starch content of the shoots were not affected by the applied water amount (Figures 3B,E). However, sucrose and raffinose in the shoots increased in 50 and 100% ET_c_ treatments compared with 25% ET_c_ (Table 4). Root carbohydrate content (Figures 3C,F) and composition were not affected by irrigation treatments, with sucrose being the main soluble sugar found in root tissues (Table 4).

**TABLE 4:** Individual sugars (mg/g of dry weight) determined in the leaves, shoots, roots, and berry must (g/L) of Cabernet Sauvignon grapevines (clone FPS08) subjected to different replacements of crop evapotranspiration (25% ET_c_, 50% ET_c_, and 100% ET_c_) during two growing seasons (2018–2019 and 2019–2020) and harvested in Oakville, CA, United States, in October 2020.

	Leaves				Shoots				Roots				Berry must			
	D-Fructose	D-Glucose	D-Sucrose	D-Raffinose	D-Fructose	D-Glucose	D-Sucrose	D-Raffinose	D-Fructose	D-Glucose	D-Sucrose	D-Raffinose	D-Fructose	D-Glucose	D-Sucrose	SS
**Treatments**																
25% ET_c_	19.17 ± 1.73^b^	25.41 ± 2.91^b^	14.32 ± 4.17	10.41 ± 0.72^b^	8.84 ± 0.81	12.59 ± 1.17	1.45 ± 0.13^b^	0.91 ± 0.10^b^	2.52 ± 0.32	3.10 ± 0.18	19.20 ± 2.31	1.88 ± 0.11	229.2 ± 55.1	348.3 ± 69.6	0.75 ± 0.10	578.3 ± 124.7
50% ET_c_	21.55 ± 1.09^ab^	24.55 ± 0.92^b^	8.69 ± 1.24	10.82 ± 0.94^b^	9.97 ± 0.74	13.25 ± 0.80	4.28 ± 0.48^a^	1.62 ± 0.22^a^	3.09 ± 0.45	3.78 ± 0.40	19.40 ± 1.48	2.29 ± 0.21	187.3 ± 36.5	246.9 ± 47.7	0.54 ± 0.10	434.7 ± 79.9
100% ET_c_	25.56 ± 2.32^a^	34.01 ± 2.89^a^	8.91 ± 1.23	14.08 ± 1.37^a^	8.58 ± 1.17	12.07 ± 1.37	3.96 ± 0.44^a^	1.93 ± 0.11^a^	2.06 ± 0.25	3.16 ± 0.37	15.30 ± 0.73	1.78 ± 0.17	179.6 ± 40.2	284.6 ± 54.2	0.65 ± 0.09	464.8 ± 94.1
ANOVA	*	*	ns	*	ns	ns	*	**	ns	ns	ns	ns	ns	ns	ns	ns

Values represent means (n = 6) separated by Duncan’s new multiple range test (p ≤ 0.05). Different letters within each column indicate significant differences as affected by the different irrigation amounts. ns, * or ** indicate non-significance or significance at 5% and 1% probability levels, respectively. DW, dried weight.

Our analysis of the different carbohydrates found in grapevine tissues indicated that starch was the main NSC in the shoots and roots, which accounted for >50% regardless of the applied water affecting their proportions (Table 5). In the leaves, starch content was the less abundant NSC, but a significant effect of irrigation treatments was observed with the 100% ET_c_ treatment reaching the highest amount. Finally, the proportions of sucrose and raffinose in the shoots decreased when water application was restricted to 25% ET_c_ (Table 5).

**TABLE 5:** Percentage of total non-structural carbohydrates (NSC) in the leaves, shoots, and roots of Cabernet Sauvignon grapevines (clone FPS08) subjected to different replacements of crop evapotranspiration (25% ET_c_, 50% ET_c_, and 100% ET_c_) during two growing seasons (2018–2019 and 2019–2020) and harvested in Oakville, CA, United States, in October 2020.

	% of total NSC				
	D-Fructose	D-Glucose	D-Sucrose	D-Raffinose	Starch
**Leaf**					
25% ET_c_	26.91 ± 1.77	35.59 ± 3.10	19.06 ± 4.86	14.58 ± 0.54	3.86 ± 0.73^c^
50% ET_c_	30.38 ± 1.34	34.61 ± 0.88	12.32 ± 1.84	15.24 ± 1.17	7.45 ± 0.91^b^
100% ET_c_	26.96 ± 1.29	35.79 ± 1.40	9.53 ± 1.38	14.80 ± 0.79	12.92 ± 1.67^a^
ANOVA	Ns	ns	ns	ns	***
**Shoot**					
25% ET_c_	15.99 ± 0.63	22.79 ± 0.80	2.69 ± 0.24^b^	1.65 ± 0.11^b^	56.89 ± 1.24
50% ET_c_	16.05 ± 0.85	21.42 ± 0.95	6.94 ± 0.76^a^	2.60 ± 0.33^a^	52.99 ± 1.24
100% ET_c_	14.45 ± 1.62	20.35 ± 1.68	6.72 ± 0.65^a^	3.31 ± 0.25^a^	55.16 ± 2.87
ANOVA	Ns	ns	***	***	ns
**Root**					
25% ET_c_	3.35 ± 0.43	4.14 ± 0.34	24.82 ± 2.01	2.52 ± 0.23	64.98 ± 2.38
50% ET_c_	3.99 ± 0.80	4.77 ± 0.61	24.11 ± 1.02	2.96 ± 0.46	64.16 ± 2.23
100% ET_c_	2.83 ± 0.37	4.31 ± 0.48	20.91 ± 0.66	2.43 ± 0.22	69.51 ± 1.14
ANOVA	Ns	ns	ns	ns	ns

Values represent means (n = 6) separated by Duncan’s new multiple range test (p ≤ 0.05). Different letters within each column indicate significant differences as affected by the different irrigation amounts. ns and *** indicate non-significance or significance at 0.1% probability levels, respectively. DW, dried weight.

Regarding the sugar composition of the must, fructose and glucose were the main sugars found (Table 4), and their ratio ranged between 0.62 and 0.78 with no difference between treatments (data not shown).

### Relationships Between the Physiological Response of Grapevine to Different Irrigation Amounts and Primary Metabolism

**FIGURE 4:**
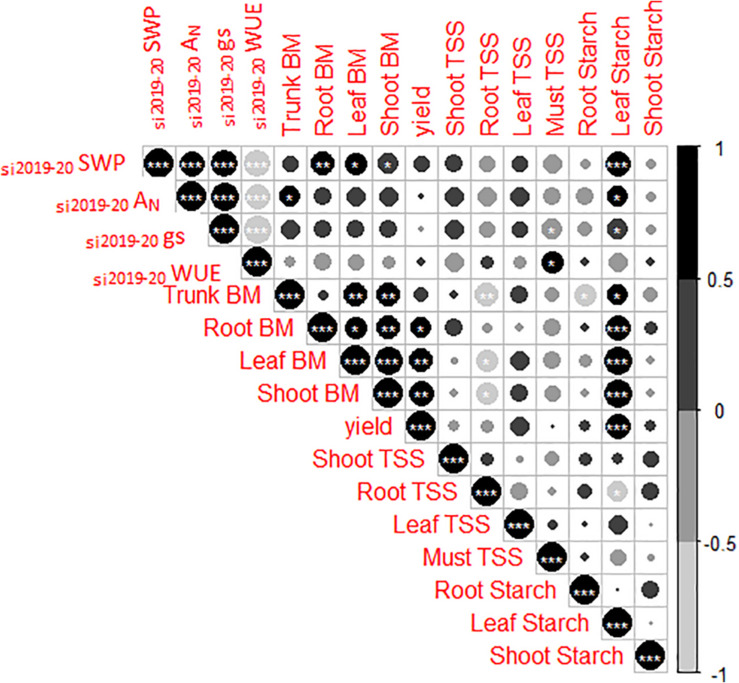
Correlation matrices among seasonal integrals for two seasons of stem water potential (_si2019–20_SWP) and gas exchange parameters (_si2019–20_*g*_s_, _si2019–20_*A*_N_, and _si2019–20_WUE), yield, vegetative growth (total biomass, BM), total soluble sugars (SS), and starch of different organs from Cabernet Sauvignon grapevines (clone FPS08), subjected to different replacements of crop evapotranspiration (25% ET_c_, 50% ET_c_, and 100% ET_c_) during two growing seasons (2018–2019 and 2019–2020) and collected in October 2020 in Oakville, CA, United States. Circle size and color represent *R* values for Pearson’s correlation analysis. *, **, and *** indicate significance at 5, 1, and 0.1% probability levels, respectively.

To analyze the carryover effect of irrigation amounts on grapevine growth and sugar metabolism, a correlation analysis was conducted (Figure 4). Thus, strong relationships between the two growing years’ seasonal integrals of SWP (_si2019–20_SWP) and gas exchange parameters (_si2019–20_*g*_s_, _si2019–20_*A*_N_, _si2019–20_WUE) were shown. A higher grapevine water status (_si2019–20_SWP) was positively related to an increased growth of roots, shoots, and leaves. Similarly, leaf starch content was strongly correlated with _si2019–20_SWP, _si2019–20_*g*_s_, and _si2019–20_*A*_N_ (*r* = 0.74 and *p* ≤ 0.0001; *r* = 0.51 and *p* ≤ 0.05; and *r* = 0.50 and *p* ≤ 0.05, respectively). On the other hand, a significant relationship between increased leaf starch content and yield per vine was observed (*r* = 0.76, *p* ≤ 0.0001). Moreover, the increases in yield per vine were related with enhancements in leaf, shoot, and root biomasses. Berry must SS were positively correlated with _si2019–20_WUE and negatively with _si2019–20_*g*_s_ (*r* = 0.55 and *p* ≤ 0.05 and *r* = -0.49 and *p* ≤ 0.05, respectively). Finally, increased vegetative growth of trunk, leaves, and shoots negatively affected root SS (*r* = -0.64 and *p* ≤ 0.01; *r* = -0.50 and *p* ≤ 0.05; and *r* = -0.57 and *p* ≤ 0.05, respectively). Conversely, positive relationships between trunk, root, leaf, and shoot biomasses with leaf starch content were recorded (*r* = 0.52 and *p* ≤ 0.05, *r* = 0.84 and *p* ≤ 0.0001; *r* = 0.79 and *p* ≤ 0.0001; and *r* = 0.88 and *p* ≤ 0.0001, respectively).

**FIGURE 5:**
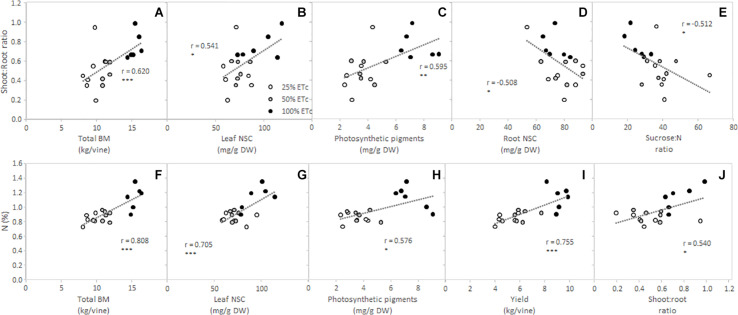
Relationship between shoot to root ratio and total biomass (BM) **(A)**, leaf non-structural carbohydrates (NSC) **(B)**, photosynthetic pigments **(C)**, root NSC **(D)**, and leaf sucrose:N ratio **(E)** and between N content and total BM **(F)**, leaf NSC **(G)**, photosynthetic pigments **(H)**, yield per vine **(I)**, and shoot to root ratio **(J)** from Cabernet Sauvignon grapevines (clone FPS08), subjected to different replacements of crop evapotranspiration (white circle, 25% ET_c_; gray circle, 50% ET_c_; and black circle, 100% ET_c_) during two growing seasons (2019 and 2020) and collected in October 2020 in Oakville, CA, United States. Dashed lines represent regression curves for Pearson’s correlation analysis. *, **, and *** indicate significance at 5, 1, and 0.1% probability levels, respectively.

Furthermore, in order to delve deeper into the effects of the replacement of different fractions of ET_c_ on grapevine physiology and metabolism, Pearson correlations between shoot:root ratio and petiole N content with the total biomass (BM) and primary metabolites were conducted (Figure 5). Shoot:root ratios of Cabernet Sauvignon vines were significantly correlated with the total BM, leaf and root NSC, photosynthetic pigments, plant sucrose to N ratio, and N contents (Figures 5A–E,J), where increased shoot:root ratio showed higher total BM, leaf NSC, chlorophylls and carotenoids, and N contents. Moreover, increased shoot to root ratio was related to decreased root NSC contents and low sucrose:N ratios (Figures 5D,E). On the other hand, the petiole N content was positively correlated with the total BM, leaf NSC, and photosynthetic pigments (Figures 5F–H), again with 100% ET_c_ grapevines reaching the highest values of all the abovementioned parameters. The petiole N content also showed a significant relationship with the yield per vine (Figure 5I).

## Discussion

### Physiological Responses of Grapevines to Different Replacements of ET_c_ Along Two Growing Seasons

In spite of the warming trends recorded for the study area within the two growing seasons covered by this study, the plant water status recorded in both growing seasons was optimal for grapevine growth as indicated by the midday SWP and the *g*_s_. Thus, seasonal integrals of SWP ranged between -0.8 and -1.1 MPa, while *g*_s_ ranged between 150 and 250 mmol m^–2^ s^–1^, in accordance to the midday SWP and *g*_s_ values considered as well-watered conditions (-0.9 MPa and 200 mmol m^–2^ s^–1^, respectively) (Medrano et al., 2002; van Leeuwen et al., 2009). Moreover, water status of the grapevines subjected to less applied water amount (25% ET_c_) never reached values lower than -1.5 MPa for SWP and/or 50 mmol m^–2^ s^–1^ for *g*_s_, which have been reported to impair grapevine performance and berry ripening (Medrano et al., 2002; Villalobos-González et al., 2019). As Keller et al. (2016) reported before, in warmer years, 100% ET_c_ treatment may suffer from mild water deficit. Thus, under our experimental conditions, at the end of the season, especially in 2020, grapevines reached SWP values to ca. -1.2 MPa; however, they are not sufficient to impair grapevine physiology and metabolism in warm climates (Romero et al., 2010). Previous studies highlighted that plant water status is closely related to leaf gas exchange parameters (Chaves et al., 2010; Zúñiga et al., 2018; Villalobos-González et al., 2019; Brillante et al., 2020). Thus, low values of SWP were related to decreased *g*_s_ likely because plants subjected to mild to moderate water deficit close their stomata as an early response to water scarcity to diminish water loss and carbon assimilation (Chaves et al., 2003).

Accordingly, in both growing seasons, a higher SWP promoted increased stomatal conductance and, consequently, net carbon assimilation rates in grapevines subjected to 100% ET_c_. *A*_N_ and *g*_s_ peaked around *veraison* and then declined in all the treatments similar to several studies conducted in a warm climate before (Romero et al., 2010; Munitz et al., 2017). Thus, previous studies have pointed out that limited photosynthetic performance, hence lower *g*_s_ and *A*_N_ values, may be triggered by passive (hydraulic signals) or active (upregulation in abscisic acid) signals (Tombesi et al., 2015). Nevertheless, *A*_N_ in 50% ET_c_ treatment was not severely decreased presumably by increases in WUE, which have been related to improvements in stomatal sensitivity to water loss and vapor pressure despite the hormonal signaling (i.e., abscisic acid) from roots to shoots (Collins et al., 2010; Medici et al., 2014). Likewise, Tortosa et al. (2019) suggested that differences in WUE between Tempranillo grapevine clones were more explanatory of the variations in carbon assimilation rather than a different stomatal control. Finally, it is worth mentioning that WUE was significantly lower in the driest and hotter growing season (i.e., 2019–2020) regardless of the irrigation treatment as previously reported (Keller et al., 2016). Regarding intrinsic WUE (*A*_N_/*g*_s_), no effect due to growing conditions was observed in contrast to previous studies on vines subjected to mild water stress (Chaves et al., 2010; Keller et al., 2016).

The water deficits applied in this study were from moderate to severe based on SWP values; thus, it is expected that the vegetative and reproductive growth of vines will be impacted accordingly. Thus, in previous studies, higher water deficits resulted in reductions of yield and berry size (Romero et al., 2010; Santesteban et al., 2011; Keller et al., 2016; Yu et al., 2016; Martínez-Lüscher et al., 2020). The reduction in berry mass has been associated with the inhibition of cell expansion (Keller, 2010) and the diminution of inner mesocarp cell sap (Roby and Matthews, 2004). The detrimental effects of 25% ET_c_ were reported previously, suggesting that this applied water amount may not be adequate for hot climates with very little or no summer precipitation (Keller et al., 2016).

Vegetative growth was also impaired by water deficits applied in this study, as indicated in the decrease of leaf and root dry biomasses measured in 25 and 50% ET_c_ treatments. Diminution of root growth under water stress has been related to the loss of cell turgor and increased penetration resistance of dried soils (Bengough et al., 2011). In addition, a recent study suggested that the loss of leaves could decrease the supply of carbohydrates and/or growth hormones to meristematic regions, thereby inhibiting growth (Karami et al., 2017). In accordance with previous studies, severe water deficits led to lower shoot to root ratio because root growth is generally less affected than shoot growth in drought-stressed grapevines (Karami et al., 2017). Given that grapevine vegetative growth occurs soon after bud break in springtime, our results corroborated the crucial role of water availability during that period on vine development, physiological performance, and yield components reported in previous studies (Munitz et al., 2020). Thus, irrigation of grapevines during summer could not be sufficient to fulfill water requirements when rainfall has been scarce in spring (Nelson et al., 2016), and precipitation amounts prior to bud break result in cascading effects for the rest of the growing season that cannot be overcome with supplemental irrigation (Martínez-Lüscher et al., 2017).

### Carbohydrate Metabolism of Grapevine Organs Responded to Different Amounts of Irrigation

The allocation of NSC varied between organs for which roots accounted 30%, shoots 25%, and leaves 40% of the whole plant NSCs at harvest, slightly differing from those reported for several fruit trees (Furze et al., 2019) but similar to the works in grapevine (Candolfi-Vasconcelos et al., 1994). The NSC composition was highly dependent on the grapevine organs, with starch being the main NSC in the roots and shoots. Previous studies reported that roots accumulated the largest amounts of starch in plastids, namely amyloplasts, which is fundamental to allow rapid vegetative development during the next spring (Zapata et al., 2004; Noronha et al., 2018). Our results also corroborated with this finding.

Our results indicated that, apart from fruits, SS were mainly accumulated in the leaves at harvest, which accounted for about 90% of the total leaf NSC. Thus, the allocation of NSCs in different organs allowed the plants to persist when respiration rate was higher than photoassimilation in annual events, but also aided in responding to abiotic stresses such as drought (Furze et al., 2019). Our results indicated that plants that received 100% ET_c_ had higher NSC content. Similarly, a previous study with potted grapevines reported increased starch and SS contents in the leaves from the grapevines with higher leaf area to fruit ratio that were well-watered (Rossouw et al., 2017b). In shoots, sucrose and raffinose proportions were higher in 50 and 100% ET_c_ treatments compared with 25% ET_c_. As a great part of the shoot biomass is vascular tissue, this may suggest an increase in NSC translocation in these treatments. Although sucrose is the main sugar for carbon translocation through the phloem into the sink tissues, recent research highlighted the roles of other sugars, such as raffinose, in carbon translocation and storage (Rossouw et al., 2017a). On the other hand, previous research reported less NSC accumulation in grapevine canes under carbon starvation at a low leaf to fruit ratios, suggesting that sucrose may control starch accumulation through adjustment of the sink strength (Silva et al., 2017). Furthermore, Rossouw et al. (2017a) also highlighted the role of raffinose (and other myo-inositol metabolism-derived metabolites) toward root carbohydrate source functioning in grapevines with significantly lower leaf to fruit ratio due to defoliation from carbon starvation (Greven et al., 2016).

When the photosynthetic supply of carbohydrates is limited, remobilization from perennial tissues can provide an alternative carbon source (Candolfi-Vasconcelos et al., 1994). Thus, previous research conducted on potted grapevines reported a concurrent starch remobilization from roots with a rapid berry sugar accumulation (Rossouw et al., 2017b). Conversely, under our experimental conditions (field-grown grapevine), no effect of water deficits on NSC remobilization from roots to berries was observed despite the decreased leaf to fruit ratio. Likewise, Keller et al. (2016) did not observe higher amounts of sugars in berries from field-grown Cabernet Sauvignon subjected to 25% ET_c_ compared with 70 or 100% ET_c_ under field conditions.

### Implications of Shoot to Root Ratio Partitioning and Nitrogen Content on Grapevine Response to Different Amounts of Irrigation

Under our experimental conditions, yield per plant was strongly related to shoot, leaf, and root BM. Similarly, Field et al. (2020) found that grapevines with the lowest shoot growth rate (elongation or dry biomass) before *veraison* had significantly less fruit set than the other treatments, attributing these effects to the restoration of root carbohydrate reserves that occurred at the same time.

Grapevines subjected to 25% ET_c_ had reduced photoassimilates due to lower *A*_N_ in both seasons resulting in less NSC in the source leaves available for new growth and exported to sinks. This resulted in a general lower plant BM (yield, leaves, shoots, and roots). Contrarily, grapevines subjected to 100% ET_c_ had higher photoassimilation rates throughout the course of the study that led to higher SS and starch content and, consequently, to the improvement of BM and, therefore, higher harvest index. Therefore, the reduced growth rate of both sink and source organs in response to water deficits indicated that the availability of carbon is a major growth constraint. The yield per plant of 50% ET_c_ was lower than 100% ET_c_, but not as low as 25% ET_c_. However, canopy BM was greatly reduced in both 50% ET_c_ and 25% ET_c_ compared with 100% ET_c_. Accordingly, Field et al. (2020) reported that grapevine grown under warm soil conditions favored shoot and fruit development over carbohydrate reserve accumulation. In contrast, Candolfi-Vasconcelos et al. (1994) reported that a lower leaf area to fruit ratio increased the translocation of carbohydrates from permanent structures to reproductive organs to support grape ripening.

The shoot to root ratio revealed a positive relationship with the total BM, leaf and root NSC, and N contents. Thus, the distribution of biomass relies on the C:N ratio as highlighted by the negative relationship between shoot to root and the sucrose:nitrogen ratios. Similarly, a linear relationship between NSC and root to shoot ratio in grapevines grown under stressful conditions was previously reported (Grechi et al., 2007). From a molecular point of view, the alterations of source:sink ratios led to transcriptional adjustments of genes involved in starch metabolism, including the upregulation of VvGPT1 and VvNTT (plastidic ATP/ADP translocator) for lower leaf area to fruit ratios (Silva et al., 2017). Furthermore, enhanced root biomass in 100% ET_c_ likely resulted from higher sugar content in the roots as our data supported. It was recently reported that increases in root elongation and hexose contents were due to the VvSWEET4 overexpression, a gene implied as a grapevine response to abiotic stress (Meteier et al., 2019). Similarly, Medici et al. (2014) reported up- or downregulation of the genes encoding hexose transporters in grapevines subjected to water deficits corroborating this result. Therefore, although some genes may be expressed under water deficit, lack of carbon accumulation impaired the growth.

The relationship between root to shoot ratio and plant nitrogen content was previously reported for grapevines, suggesting that dry matter partitioning is largely a function of the internal status of the plants (Grechi et al., 2007). We found decreased N content in grapevines facing water deficits, which resulted in a decrease of total BM. Similarly, Romero et al. (2010) reported reductions in leaf nitrogen content when vines were subjected to water deficits. These authors suggested that nutrient uptake may be reduced due to deficits in soil water profile, and the slow root growth under these conditions consequently inhibited grapevine growth.

In our study, N content was strongly related to photosynthetic pigments. Accordingly, previous studies reported lower leaf N and leaf chlorophyll in deficit-irrigated grapevines, suggesting quantitative losses in the photosynthetic apparatus and/or damage to the biochemical photosynthetic machinery, decreasing photosynthetic capacity (Romero et al., 2010) as corroborated with the lower NSC leaf content with water deficits. Finally, molecular research over the last decades has suggested the important regulatory functions of sucrose and N metabolites (i.e., amino acids) in metabolism at the cellular and subcellular levels and/or in gene expression patterns, giving new insights into how plants may modulate over a longer period its growth and biomass allocation in response to fluctuating environmental conditions (Tognetti et al., 2013).

## Conclusion

Our results provided evidence that differences in carbon assimilation and partitioning between the source and sink organs largely explained the response of grapevines to water deficits as it would be scheduled by irrigators and water purveyors in hot climates. Therefore, grapevines supplied with 25% ET_c_ showed a reduced rate of net photosynthesis, lower water status, less photoassimilates in the source (leaves) available for new growth and exported to sinks, and a lower plant BM due to water restriction. Conversely, 100% ET_c_ showed the highest photosynthetic performance and water status, which led to increased contents of soluble sugars and starch in the leaves and greater yield. However, 100% ET_c_ showed clear signs of being excessive as leaf biomass increased disproportionately to the point that it had a higher leaf area to fruit ratios. Our data revealed that in the 50% ET_c_ treatment, the enhancement of sugar transport, mainly sucrose and raffinose, could slow down the detrimental effect of water deficit on yield. Finally, an important role of sugar and nitrogen was suggested due to their significant relationship with biomass partitioning.

## Data Availability Statement

The raw data supporting the conclusions of this article will be made available by the authors, without undue reservation.

## Author Contributions

SK conceptualized, designed, and acquired the funding. EK, JM-L, NT, and RY executed and curated the data. NT and RY wrote the first version of the manuscript. All the authors read and approved the manuscript.

## Conflict of Interest

The authors declare that the research was conducted in the absence of any commercial or financial relationships that could be construed as a potential conflict of interest.

## Supplementary Material

Supplementary Table 1 Petiole mineral content of Cabernet Sauvignon grapevines (clone Fps08) subjected to different replacement of crop evapotranspiration (25% Et_c_, 50% Et_c_ and 100% Et_c_), collected in Oakville, Ca, United States in the 2018–2019 and 2019–2020 seasons.

Supplementary Table 2 Total biomass (Fw) of trunks, leaves, shoots and roots (kg/vine) of Cabernet Sauvignon grapevines (clone Fps08) subjected to different replacement of crop evapotranspiration (25% Et_c_, 50% Et_c_ and 100% Et_c_) during two growing seasons (2018-19 and 2019-20) and harvested in Oakville, Ca in November 2019 and October 2020, respectively.
