# Supplementary material for: Application of Fractions of Crop Evapotranspiration Affects Carbon Partitioning of Grapevine Differentially in a Hot Climate
Source: Front Plant Sci. 2021 Feb 22;12:633600. doi: 10.3389/fpls.2021.633600 (PMC7938324; doi:10.3389/fpls.2021.633600)
Supplement: Supplementary Table 1 — Petiole mineral content of Cabernet Sauvignon grapevines (clone Fps08) subjected to different replacement of crop evapotranspiration (25% Etc, 50% Etc and 100% Etc), collected in Oakville, Ca, United States in the 2018–2019 and 2019–2020 seasons. [file Data_Sheet_1.docx]

**Table S1**. Petiole mineral content of Cabernet Sauvignon grapevines (clone FPS08) subjected to different replacement of crop evapotranspiration (25% ET_c_, 50% ET_c_ and 100% ET_c_), collected in Oakville, CA in 2018-19 and 2019-20 seasons.

|  |  | N | P | K | Zn | Mn | Na | Ca | B | Mg | Fe | Cu |
| --- | --- | --- | --- | --- | --- | --- | --- | --- | --- | --- | --- | --- |
|  |  | % | % | % | mg/kg | mg/kg | % | mg/kg | % | % | mg/kg | mg/kg |
| 2019 | | |  |  |  |  |  |  |  |  |  |  |
| Treatments | |  |  |  |  |  |  |  |  |  |  |  |
|  | 25% ET_c_ | 0.69 ± 0.02 | 0.53 ± 0.04 | 1.25 ± 0.07 | 113.0 ± 1.5 | 31.7 ± 2.3 | 0.018 ± 0.003 | 2.23 ± 0.19 | 68.00 ± 1.52 | 0.69 ± 0.03 | 30.67 ± 2.60 | 8.67 ± 0.34 |
|  | 50% ET_c_ | 0.69 ± 0.02 | 0.59 ± 0.01 | 1.27 ± 0.01 | 119.7 ± 3.9 | 30.3 ± 0.9 | 0.010 ± 0.004 | 2.38 ± 0.09 | 67.77 ± 1.67 | 0.68 ± 0.03 | 31.67 ± 1.86 | 9.33 ± 0.34 |
|  | 100% ET_c_ | 0.73 ± 0.03 | 0.59 ± 0.02 | 1.41 ± 0.13 | 121.0 ± 7.2 | 30.7 ± 1.8 | 0.013 ± 0.003 | 2.45 ± 0.13 | 69.00 ± 1.53 | 0.68 ± 0.01 | 30.67 ± 1.45 | 10.00 ± 0.57 |
|  |  |  |  |  |  |  |  |  |  |  |  |  |
| *ANOVA* | | ns | ns | ns | ns | ns | ns | ns | ns | ns | ns | ns |
| 2020 | |  |  |  |  |  |  |  |  |  |  |  |
| Treatments | |  |  |  |  |  |  |  |  |  |  |  |
|  | 25% ET_c_ | 0.82 ± 0.03 b | 0.52 ± 0.03 | 2.92 ± 0.15 b | 101.0 ± 2.0 | 36.8 ± 2.8 | 0.007 ± 0.002 | 1.73 ± 0.14 | 44.20 ± 0.70 | 0.68 ± 0.05 | 36.00 ± 1.91 | 12.00 ± 0.63 |
|  | 50% ET_c_ | 0.90 ± 0.02 b | 0.49 ± 0.02 | 3.11 ± 0.10 a | 99.8 ± 2.0 | 36.0 ± 1.5 | 0.008 ± 0.002 | 1.89 ± 0.10 | 46.50 ± 1.18 | 0.68 ± 0.03 | 33.67 ± 2.22 | 12.17 ± 0.54 |
|  | 100% ET_c_ | 1.18 ± 0.06 a | 0.46 ± 0.02 | 3.36 ± 0.19 a | 94.4 ± 6.2 | 39.2 ± 4.6 | 0.006 ± 0.002 | 1.73 ± 0.09 | 47.80 ± 2.92 | 0.64 ± 0.07 | 36.60 ± 2.64 | 11.40 ± 0.75 |
|  | |  |  |  |  |  |  |  |  |  |  |  |
| *ANOVA* | | *** | ns | * | ns | ns | ns | ns | ns | ns | ns | ns |
|  |  |  |  |  |  |  |  |  |  |  |  |  |

Values represent means (n = 6) separated by Duncan’s new multiple range test (P≤ 0.05). Different letters within each column, indicate significant differences as affected by the irrigation amounts. ns, *, and *** indicate non-significance or significance at 5% and 0.1% probability levels, respectively. Values are expressed as % or mg of the mineral per kg of petiole dry weight.

**Table S2**. Total biomass (FW) of trunks, leaves, shoots and roots (kg/vine) of Cabernet Sauvignon grapevines (clone FPS08) subjected to different replacement of crop evapotranspiration (25% ET_c_, 50% ET_c_ and 100% ET_c_) during two growing season (2018-19 and 2019-20) and harvested in Oakville, CA in November 2019 and October 2020, respectively.

|  |  | Leaves | Shoots | Roots |
| --- | --- | --- | --- | --- |
|  |  |  |  |  |
| 2019 |  |  |  |  |
| Treatments | |  |  |  |
|  | 25% ET_c_ | 1.30 ± 0.04 b | 1.22 ± 0.10 b | ND |
|  | 50% ET_c_ | 1.99 ± 0.20 a | 1.56 ± 0.17 ab | ND |
|  | 100% ET_c_ | 2.55 ± 0.09 a | 1.89 ± 0.19 a | ND |
|  |  |  |  |  |
| *ANOVA* | | ** | ** |  |
| 2020 |  |  |  |  |
| Treatments | |  |  |  |
|  | 25% ET_c_ | 1.26 ± 0.17 b | 0.71 ± 0.08 b | 1.92 ± 0.20 b |
|  | 50% ET_c_ | 1.42 ± 0.10 b | 0.98 ± 0.10 b | 2.24 ± 0.11 ab |
|  | 100% ET_c_ | 2.47 ± 0.22 a | 1.74 ± 0.17 a | 2.64 ± 0.21 a |
|  |  |  |  |  |
| *ANOVA* | | *** | *** | * |
|  |  |  |  |  |

Values represent means (n = 6) separated by Duncan’s new multiple range test (P≤ 0.05). Different letters within each column, indicate significant differences as affected by the irrigation amounts. ns, *, ** and *** indicate non-significance or significance at 5%, 1% and 0.1% probability levels, respectively. ND, non-determined.
